# Machine learning–based identification of multidimensional predictors of quality of life in individuals with multiple sclerosis

**DOI:** 10.1097/MD.0000000000049025

**Published:** 2026-05-29

**Authors:** Aysu Yetiş, Mehmet Canli, İrem Canli, Hikmet Kocaman, Hasan Yildirim, Nazim Tolgahan Yildiz, Selcen Duran

**Affiliations:** a Faculty of Medicine, Department of Neurology, Kirşehir Ahi Evran University, Kirşehir, Turkey; b School of Physical Therapy and Rehabilitation, Kirşehir Ahi Evran University, Kirşehir, Turkey; c Faculty of Health Sciences, Deparment of Physiotherapy and Rehabilitation, Karamanoglu Mehmetbey University, Karaman, Turkey; d Faculty of Kamil Özdağ Science, Department of Mathematics, Karamanoğlu Mehmetbey University, Karaman, Turkey.

**Keywords:** disability, machine learning, multiple sclerosis, quality of life

## Abstract

This study aimed to identify independent predictors of quality of life in patients with multiple sclerosis (MS) using machine learning approaches. One hundred and one individuals diagnosed with MS were included in this cross-sectional study. Quality of life was assessed using the MS Quality of Life-54 (MSQoL-54). Demographic variables, clinical characteristics, disability level (Expanded Disability Status Scale [EDSS]), fatigue severity, sleep quality (Pittsburgh Sleep Quality Index), depression level (Beck Depression Inventory), and functional mobility (Timed Up and Go test) were evaluated. Multiple machine learning regression models, including Linear Regression, Lasso, Elastic Net, Support Vector Machines, Random Forest, and XGBoost, were developed and compared. Five-times repeated five-fold cross-validation was applied for internal validation. Model performance was evaluated using root mean squared error (RMSE), mean absolute error (MAE), and coefficient of determination (*R*^2^). Among all models, Lasso regression demonstrated the best predictive performance (RMSE = 5.02, MAE = 4.04, *R*^2^ = 0.86). Disability level (EDSS) emerged as the strongest negative predictor of quality of life, followed by fatigue severity, sleep quality, and impaired functional mobility. Although disease duration was not significantly correlated with quality of life in univariate analyses, it showed a positive contribution in the multivariable model. Depression showed a strong bivariate association but had a relatively lower weight in the final predictive model. Quality of life in individuals with MS is strongly influenced by disability, fatigue, sleep quality, and functional mobility. Machine learning approaches provide an effective and interpretable framework for identifying key determinants of quality of life and may support personalized rehabilitation and clinical decision-making in MS.

## 1. Introduction

Multiple sclerosis (MS) is one of the most common neurological diseases in young adults, affecting approximately 2.3 million people worldwide. The disease has the highest prevalence among those aged 20 to 40, who are considered economically active.^[1]^ It is considered the second most debilitating chronic disease of the central nervous system after stroke and before Parkinson disease.^[2,3]^ The clinical symptoms of MS include coordination and balance disorders, muscle spasms, fatigue, pain and vision problems; these symptoms can lead to restricted mobility and, in some cases, require hospitalization.^[4,5]^

MS is one of the most debilitating diseases seen in young people and has multifaceted negative effects on patients’ quality of life. Individuals living with chronic and progressive diseases such as MS experience significant losses in physical, social and cognitive functioning, which leads to a decline in their quality of life.^[6]^ Quality of life is a multidimensional concept defined by the World Health Organisation as individuals’ perceptions of their position in life in the light of the cultural context, value systems, goals, expectations, standards and concerns within which they live.^[7]^ Low quality of life increases stress levels and contributes to the severity of the disease by leading to ineffective coping and adaptation mechanisms.^[8]^

Quality of life in MS is determined by numerous physical and psychosocial factors. The severity and duration of the disease, weakness, disability and walking difficulties are important predictors of quality of life.^[9]^ Psychological problems in women and physical functional limitations in men have a more pronounced effect on quality of life.^[10]^ In the literature, it has been demonstrated that psychological and physical symptoms such as depression, anxiety, fatigue, sleep disorders, pain, and sexual dysfunction negatively affect patients’ adherence to treatment and their active participation in the process.^[11–14]^ In a study conducted on early-stage MS patients, it was reported that low quality of life scores were associated with greater fatigue, sleep problems, depression and anxiety; furthermore, these patients had lower self-efficacy, internal locus of control and social support, and higher levels of neuroticism.^[15]^

In recent years, machine learning approaches have been increasingly utilized in health research due to their potential to reveal complex and non-linear relationships between numerous variables. Unlike traditional statistical methods, machine learning algorithms enable more accurate and robust predictions at an individual level by learning patterns from large and multidimensional datasets.^[16,17]^ Particularly in chronic diseases with a heterogeneous clinical course, such as MS, machine learning-based models can contribute to the development of personalized approaches by enabling the joint assessment of clinical and psychosocial variables in identifying factors that affect quality of life.

Although previous studies have investigated determinants of quality of life in individuals with MS, several gaps remain. Most studies have used traditional statistical methods and have generally examined clinical, functional, or psychosocial factors separately. As a result, the combined and relative predictive contributions of disability, fatigue, sleep quality, depression, mobility, and demographic characteristics remain insufficiently understood. Moreover, machine learning approaches have rarely been applied to predict quality of life in MS, and comparative evaluations of multiple algorithms with interpretable variable importance analysis are limited. Therefore, the present study aimed to address this gap by integrating multidimensional predictors into a machine learning framework to identify the most influential determinants of quality of life in individuals with MS.

## 2. Materials and methods

### 2.1. Study design and ethical aspects

This cross-sectional study was approved by the Scientific Research Ethics Committee of Kirşehir Ahi Evran University (No: 2024-09/64). The study was conducted in accordance with the Declaration of Helsinki, and written and verbal informed consent was obtained from participants diagnosed with MS.

### 2.2. Participants

Participants were selected from individuals who presented to the Neurology Clinic at Kirşehir Training and Research Hospital and volunteered to participate in the study. The study population consisted of patients diagnosed with MS by a specialist neurologist and whose native language was Turkish. The inclusion criteria for the study were as follows: being aged between 18 and 65, having an Expanded Disability Status Scale (EDSS) score between 1 and 4, and having been diagnosed with MS by a specialist neurologist according to the McDonald criteria.^[18]^ Individuals who had experienced a flare-up of symptoms in the last 3 months, who had a Mini-Mental State Examination score below 24 indicating cognitive impairment.^[19]^ who had any non-MS neurological or musculoskeletal disorder that could adversely affect walking and balance, who had uncorrected visual or hearing impairment, or who were pregnant were excluded from the study.

### 2.3. Measurements

Participants’ sociodemographic data, such as age, BMI, gender, MS type, and disease type, were collected through face-to-face interviews. The quality of life, disability level, fatigue severity, sleep quality, and mobility level of participants with MS were assessed by the same researcher (AY) who conducted the interviews.

### 2.4. Outcome measurement

#### 2.4.1. Quality of life

Participants’ quality of life was assessed using the MS Quality of Life-54 (MSQoL-54) questionnaire. The MSQoL-54 is a comprehensive measurement tool comprising 54 items, developed to measure quality of life in individuals with MS.^[20]^ The scale comprises a 36-item short form (SF-36) that assesses general health status and quality of life, along with 18 additional items specific to multiple sclerosis. The MSQoL-54 examines health status across 2 main dimensions: physical health (39 items) and mental health (15 items). Scores obtained from the physical and mental health dimensions range from 0 to 100, with higher scores indicating better quality of life. The scale’s overall quality of life score is calculated by taking the average of the total scores obtained from these 2 subscales.^[20]^

#### 2.4.2. Disability

EDSS was used to assess patients’ disability levels. Developed by Kurtzke^[21]^ the EDSS is an assessment tool that quantitatively measures disability in MS, with a score of 0 indicating a normal neurological examination and a score of 10 indicating death due to MS.^[21]^

#### 2.4.3. Fatigue severity

The Fatigue Severity Scale (FSS) used in the study to assess fatigue severity consists of a total of 9 items aimed at determining participants’ fatigue levels over the past week.^[22]^ Each item is rated on a Likert-type scale ranging from “0 = strongly disagree” to “7 = strongly agree,” and the sum of the scores reflects the individual’s fatigue level. The maximum possible score on the scale is 63, with higher scores indicating more severe fatigue. According to the threshold value generally accepted in the literature, scores of 36 and above indicate the presence of clinically significant fatigue. In this respect, the scale is used as a reliable measurement tool for objectively determining fatigue in MS and similar chronic diseases.^[22]^

#### 2.4.4. Sleep quality

The Pittsburgh Sleep Quality Index (PSQI), developed by Buysse et al^[23]^ in 1989, is a reliable and valid measure of sleep quality for the Turkish population.^[24]^ The PSQI is the most commonly used general measure for assessing sleep quality in clinical and research settings. PSQI consists of a total of 24 questions. The questions on the scale are designed to assess sleep duration, sleep latency, and the frequency and severity of specific sleep-related problems. The first 18 items on the scale are grouped into 7 component scores. Based on the scale result, individuals are positioned somewhere between those with good sleep quality and those with poor sleep quality. The scale consists of a total of 21 points, and the higher the score an individual receives on the scale, the poorer their sleep quality is interpreted to be.^[24]^

#### 2.4.5. Mobility

The Timed Up and Go Test (TUG) is a test designed to measure functional mobility, balance, walking ability, and risk of falling. In this test, individuals are instructed to stand up as quickly and safely as possible, walk to a cone 3 meters away, turn around it, walk back, and sit down on a chair. The time taken to complete this performance is measured to determine the individual’s functional mobility. The test is performed 3 times, and the average time is calculated.^[25]^

#### 2.4.6. Depression

The Beck Depression Inventory (BDI) was developed by Beck et al^[26]^ to assess the severity of depressive symptoms in individuals, and the validity and reliability of its Turkish version have been confirmed by studies.^[27]^ The scale consists of a total of 21 items, each with 4 response options for self-assessment. Each item is scored between 0 and 3 points. The total score obtainable from the scale ranges from 0 to 63, with higher scores indicating an increased level of depressive symptoms or severity of depression.^[27]^

### 2.5. Statistical analysis

Descriptive statistics were calculated to summarize the characteristics of the study population. Continuous variables were presented as mean and standard deviation, depending on their distribution. Categorical variables were reported as counts (*n*) and percentages (%). All statistical analyses and model development were performed using MedCalc Statistical Software (version 23.0.9; MedCalc Software Ltd, Ostend, Belgium) and R (version 4.5.1; R Foundation for Statistical Computing, Vienna, Austria). A two-sided *P*-value of <.05 was considered to indicate statistical significance for all inferential tests. Given the exploratory nature of the univariate analyses, no formal correction for multiple comparisons was applied. Therefore, the results of the univariate analyses were interpreted as preliminary and hypothesis-generating rather than confirmatory. The primary emphasis of the study was placed on the cross-validated multivariable machine learning models and the relative importance of predictors in the final model.

#### 2.5.1. Outcome and predictor variables

The primary outcome for this study was the total quality of life score, as measured by the MSQoL-54 instrument. This continuous variable served as the target for all regression models. A set of predictor variables, selected based on clinical relevance and prior literature, were used for model development. These included: demographic variables (age, sex), an anthropometric variable (BMI), clinical indicators of disease status (EDSS score, disease duration), and scores from functional and psychological assessments (TUG test, PSQI, BDI score, and FSS). The MS subtype variable was excluded from the final predictor set to create a more generalizable model applicable across different clinical presentations. The individual physical and mental component scores of the MSQoL-54 were also excluded to prevent data leakage and target variable collinearity.

#### 2.5.2. Data preprocessing

Prior to model development, the dataset was examined for missing values across all outcome and predictor variables. No missing data were identified in the final dataset; therefore, no imputation procedure was required. All participants included in the analysis had complete data for the MSQoL-54 total score and all predictor variables used in the machine learning models.

A comprehensive and reproducible preprocessing pipeline was constructed to prepare the data for modeling. To ensure that no data leakage occurred from the validation sets into the training process, all preprocessing transformations were defined as a single sequence of steps that was fitted exclusively within each cross-validation fold. This pipeline began by converting nominal categorical predictors into a numeric format using one-hot encoding. Subsequently, any predictors with zero or near-zero variance, which provide little to no predictive information, were removed from the dataset. Finally, all remaining numeric predictors were standardized by centering them to have a mean of zero and scaling them to have a standard deviation of one. This standardization step ensures that variables with larger original scales do not disproportionately influence the training of the models.

#### 2.5.3. Machine learning model development and validation

To identify the most effective predictive algorithm, a wide array of regression models was systematically developed and evaluated. The analysis was conducted in R utilizing the tidymodels ecosystem, with specific model engines sourced from packages such as glmnet, ranger, xgboost, kernlab, and earth. The candidate algorithms included a standard multiple linear regression to serve as a baseline, as well as more complex models such as Random Forest, Gradient Boosting Machine (XGBoost), Support Vector Machines (with Linear, Polynomial, and Radial Basis Function kernels), and multivariate adaptive regression splines. Given the potential for high collinearity among clinical predictors, regularized regression methods including Lasso and Elastic Net were specifically included for their ability to handle multicollinearity by performing automated variable selection and coefficient shrinkage, thereby producing more stable and interpretable models.

Five times repeated five-fold cross-validation was employed to provide a stable and unbiased estimate of model performance and to guide hyperparameter tuning. This approach involves repeatedly partitioning the dataset into 5 folds (with 5 different random seeds), which helps to mitigate the risk of performance estimates being influenced by a single, potentially biased, partitioning of the dataset. For models with tunable hyperparameters, a grid search was performed across forty different hyperparameter combinations for each algorithm. The optimal combination for each model was selected based on the one that minimized the root mean squared error (RMSE), which was chosen as the primary performance metric for model selection.

#### 2.5.4. Model performance evaluation and interpretability

The performance of each optimized model was comprehensively evaluated across the cross-validation folds using a set of standard regression metrics. The primary metric for model comparison was the RMSE, which quantifies the average magnitude of prediction error in the original units of the quality of life score. To further assess the models, we also calculated the coefficient of determination (*R*^2^) to evaluate the proportion of variance in the quality of life score explained by the predictors, and the mean absolute error (MAE) to provide a linear and easily interpretable measure of average error magnitude. The model demonstrating the best overall performance, primarily determined by the lowest mean RMSE, was selected as the final model. To enhance transparency and understand the drivers of its predictions, this final model was fitted on the entire dataset using its optimal hyperparameters. For this best-performing model, variable importance was assessed by examining the final standardized coefficients. A variable importance plot was generated to rank the predictors, where importance was defined as the absolute magnitude of the coefficient, and the sign of the coefficient was used to indicate the direction of the effect.

## 3. Results

The baseline demographic and clinical characteristics of the 101 participants included in the study are summarized in Table 1. The cohort had a mean age of 46.43 ± 6.76 years and was predominantly female (*n* = 63, 62.38%). The average disease duration of participants was 12.29 ± 3.91 years, while the average EDSS scores were 2.87 ± 1.58. The primary outcome, the total MSQoL-54 score, had a mean of 59.17 ± 13.15.

**Table 1:** Baseline demographic and clinical characteristics of the study participants (n = 101).

Quantitative variables		Mean	SD
Age (yr)		46.43	6.76
BMI (kg/m^2^)		25.55	2.00
Disease duration		12.29	3.91
MSQoL 54 physical health composite		60.39	13.15
MSQoL 54 mental health composite		57.94	13.68
MSQoL 54 total score		59.17	13.15
EDSS score		2.87	1.58
BDI score		26.53	10.46
FSS score		30.51	9.71
PSQI		11.18	3.73
TUG test time (s)		11.92	1.90
**Qualitative variables**		**Count**	**Percentage (%**)
Sex	Male	38	37.62
	Female	63	62.38
MS type	RR	72	71.29
	SP	20	19.80
	PP	9	8.91

BDI = Beck Depression Inventory, BMI = body mass index, EDSS = expanded disability status scale, FSS = Fatigue Severity Scale, MS = multiple sclerosis, MSQoL-54 = Multiple Sclerosis Quality of Life-54, PP = primer progressive, PSQI = Pittsburgh Sleep Quality Index, RR = relapsing–remitting, SD = standard deviation, SP = secondary progressive, TUG = Timed Up and Go Test.

Exploratory univariate analyses were conducted to examine preliminary relationships between predictor variables and quality of life scores (Tables 2 and 3). No statistically significant differences in total MSQoL-54 scores were observed between male and female participants (*P* = .481) or across the different MS subtypes (*P* = .637), suggesting that these categorical variables were not primary drivers of quality of life in univariate comparisons.

**Table 2:** Bivariate analysis of quality of life scores across participant subgroups.

	MSQoL-54 (physical health) score				MSQoL-54 (mental health) score				MSQoL-54 (Total) score			
	Mean	SD	*P*	ES	Mean	SD	*P*	ES	Mean	SD	*P*	ES
Sex												
Male	61.74	13.11	.433	−0.129	58.91	14.06	.528	−0.104	60.33	13.36	.481	−0.116
Female	59.29	13.31			57.14	13.57			58.21	13.14		
MS type												
RR	58.67	10.08	.627	−0.019	55.67	11.78	.681	0.015	57.17	10.77	.637	0.018
SP	60.91	14.87			58.94	15.04			59.92	14.65		
PP	63.33	5.77			58.33	5.77			60.83	5.77		

ES = effect size, MSQoL-54 = Multiple Sclerosis Quality of Life-54, PP = primer progressive, RR = relapsing–remitting, SD = standard deviation, SP = secondary progressive.

**Table 3:** Correlation matrix of predictor variables and quality of life outcomes.

	MSQoL-54 (physical)			MSQoL-54 (mental)			MSQoL-54 (total)		
	rho (*P*)	95% CI	ES	rho (*P*)	95% CI	ES	rho (*P*)	95% CI	ES
Age (yr)	0.020 (.890)	[-0.257, 0.294]	0.020	0.020 (.890)	[−0.257, 0.294]	0.020	0.023 (.874)	[−0.254, 0.296]	0.023
BMI (kg/m^2^)	−0.015 (.918)	[−0.289, 0.262]	−0.015	−0.045 (.600)	[−0.344, 0.205]	−0.075	−0.036 (.697)	[−0.326, 0.223]	−0.056
Disease duration (years)	0.214 (.131)	[−0.065, 0.463]	0.218	0.130 (.364)	[−0.151, 0.391]	0.130	0.182 (.202)	[−0.099, 0.436]	0.184
EDSS score	−0.813 **(<.001**)	[−0.890, −0.693]	−1.137	−0.891 (<.001)	[−0.936, −0.815]	−1.426	−0.876 (<.001)	[−0.927, −0.791]	−1.356
BDI score	−0.682 **(<.001**)	[−0.806, −0.501]	−0.833	−0.747 (<.001)	[−0.848, −0.594]	−0.967	−0.729 (**<.001**)	[−0.837, −0.568]	−0.927
FSS score	−0.700 **(<.001**)	[−0.818, −0.525]	−0.867	−0.788 (<.001)	[−0.874, −0.654]	−1.066	−0.765 (**<.001**)	[−0.860, −0.620]	−1.009
PSQI score	−0.734 **(<.001**)	[−0.839, −0.574]	−0.937	−0.748 (<.001)	[−0.848, −0.594]	−0.967	−0.761 (**<.001**)	[−0.857, −0.615]	−0.999
TUG test time (s)	−0.494 **(<.001**)	[−0.677, −0.252]	−0.541	−0.451 (<.001)	[−0.646, −0.200]	−0.485	−0.495 (**<.001**)	[−0.678, −0.254]	−0.542

BDI = Beck Depression Inventory, BMI = body mass index, CI = confidence interval, EDSS = Expanded Disability Status Scale, ES = effect size, FSS = Fatigue Severity Scale, MS = multiple sclerosis, MSQoL-54 = Multiple Sclerosis Quality of Life-54, PSQI = Pittsburgh Sleep Quality Index, TUG = Timed Up and Go Test.

In contrast, Spearman rank correlation analysis revealed several strong and statistically significant associations with the total MSQoL-54 score (Table 3). The strongest correlation was observed with the EDSS score, which showed a very strong negative relationship with quality of life (ρ = −0.876, *P* < .001). Similarly, strong to moderate negative correlations were found for FSS (ρ = −0.765, *P* < .001), PSQI (ρ = −0.761, *P* < .001), BDI (ρ = −0.729, *P* < .001), and impaired TUG (ρ = −0.495, *P* < .001). Conversely, disease duration did not show a statistically significant correlation with the total quality of life score in this bivariate analysis (ρ = 0.182, *P* = .202).

A comprehensive evaluation of 9 machine learning models was performed to identify the algorithm with the highest predictive accuracy for the total MSQoL-54 score. As detailed in Table 4, the regularized linear models demonstrated superior performance compared to more complex, non-linear algorithms. The predictive performance of the evaluated models is presented in Table 4. Among all models, Lasso regression achieved the best overall performance, with the lowest MAE (4.0387) and RMSE (5.0226) values and the highest coefficient of determination (*R*^2^ = 0.8637). Elastic Net demonstrated the second-best performance (MAE = 4.2111, RMSE = 5.2158, *R*^2^ = 0.8323), followed by SVM with a linear kernel (MAE = 4.3354, RMSE = 5.3727, *R*^2^ = 0.8210). Models with greater structural complexity, such as Random Forest and XGBoost, showed lower predictive accuracy, with higher error values and lower explained variance. Conventional linear regression exhibited the weakest performance among the evaluated models (MAE = 5.8370, RMSE = 6.9486, *R*^2^ = 0.7149).

**Table 4:** Performance metrics and optimal hyperparameters of machine learning models for Predicting MSQoL-54 total score.

Model	MAE (95% CI)	RMSE (95% CI)	*R*^2^ (95% CI)	Hyperparameters
Lasso	4.0387 (3.72–4.36)	5.0226 (4.61–5.44)	0.8637 (0.821–0.906)	Regularization penalty (λ) = 0.07872
Elastic net	4.2111 (3.88–4.55)	5.2158 (4.79–5.64)	0.8323 (0.785–0.880)	Regularization penalty (λ) = 0.05223, L1/L2 mixture ratio (α) = 0.92692
SVM linear	4.3354 (3.99–4.68)	5.3727 (4.93–5.82)	0.8210 (0.770–0.872)	Cost (c) = 0.66577
SVM polynomial	4.4112 (4.05–4.77)	5.5663 (5.10–6.03)	0.8119 (0.758–0.866)	Cost (c) = 0.76598, polynomial degree (d) = 1
MARS	4.6241 (4.24–5.02)	5.7875 (5.29–6.28)	0.8017 (0.744–0.859)	Number of terms = 5, interaction degree = 1
SVM radial	4.8028 (4.39–5.22)	6.1077 (5.58–6.64)	0.7884 (0.724–0.853)	Cost (c) = 24.511, sigma (σ) = 0.00888
Random forest	4.9105 (4.48–5.34)	6.1391 (5.60–6.68)	0.7706 (0.701–0.840)	Predictors per split (mtry) = 6, min. node size (min_n) = 3
XGBoost	5.2880 (4.82–5.75)	6.3602 (5.79–6.93)	0.7588 (0.686–0.832)	Minimum node size (min_n) = 2, maximum tree depth = 5, learning rate (η) = 0.05298
Linear regression	5.8370 (5.29–6.38)	6.9486 (6.31–7.59)	0.7149 (0.632–0.798)	–

CI = confidence interval, MAE = Mean Absolute Error, MARS = Multivariate Adaptive Regression Splines, MSQoL-54 = Multiple Sclerosis Quality of Life-54, *R*^2^ = coefficient of determination, RMSE = Root Mean Squared Error, SVM = support vector machines, XGBoost = gradient boosting machine.

**Figure 1.**
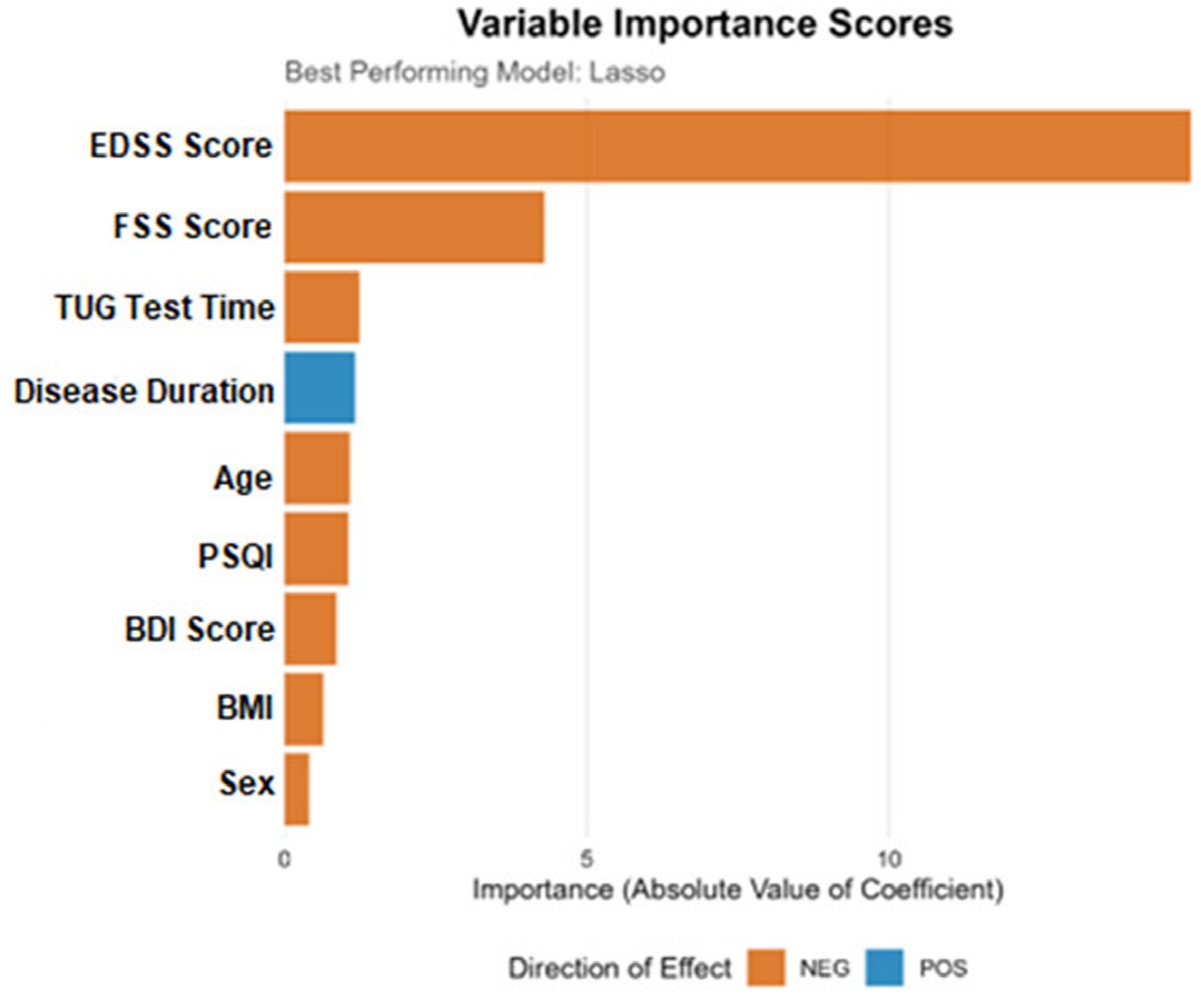
Variable importance scores derived from the best-performing Lasso regression model for predicting the MSQoL-54 total score. Variable importance was calculated using the absolute values of the standardized regression coefficients from the final Lasso model. Longer bars indicate greater relative contribution of the predictor to the model. The direction of effect is represented by color: orange bars indicate negative associations with quality of life, whereas blue bars indicate positive associations. EDSS showed the highest relative importance, followed by FSS and TUG test time. BDI = Beck Depression Inventory, BMI = body mass index, EDSS = Expanded Disability Status Scale, FSS = Fatigue Severity Scale, PSQI = Pittsburgh Sleep Quality Index, TUG = Timed Up and Go.

The final Lasso model was used to determine the relative importance and direction of effect for each predictor, as illustrated in the variable importance plot (Fig. 1). The EDSS was decisively the most influential predictor, exhibiting a strong negative association with quality of life. Consistent with clinical expectations, the FSS score was identified as the second most important predictor, also showing a clear negative impact. In contrast, while disease duration showed no significant bivariate correlation with quality of life, it emerged as the third most important variable in the multivariable model but with a positive direction of effect. This counter-intuitive finding for disease duration may be attributable to the complex interplay of variables within the model, where long-term patient adaptation or survivorship bias could influence outcomes after adjusting for the primary impact of disability. Other predictors with a notable negative impact on quality of life included TUG time, age, PSQI, BMI, and female sex. The BDI score had a comparatively smaller influence on the final model’s predictions.

## 4. Discussion

The primary objective of this study was to identify independent determinants affecting quality of life in patients with MS using machine learning approaches. The most important finding of the study is that regularized regression models, particularly the Lasso algorithm, provide high accuracy in predicting quality of life and highlight clinically meaningful variables. The results obtained show that quality of life in MS is a multidimensional construct and is closely related to disability, fatigue and mobility in particular.

In current study, the EDSS score was identified as the strongest negative predictor of quality of life. This finding is largely consistent with previous studies. The literature reports that EDSS has a decisive effect on both physical and mental quality of life components; increasing disability levels are associated with loss of independence, reduced social participation, and psychological burden.^[2,28,29]^ Our results reinforce these findings by demonstrating that EDSS is a dominant predictor not only in binary or univariate analyses but also in multivariate and adjusted models.

In the present study, fatigue severity has emerged as the second most important factor affecting quality of life. It is known that fatigue in MS is a symptom that can be observed from the early stages of the disease and severely restricts daily living activities.^[11,29]^ In our study, the fact that FSS scores showed a strong negative effect in both correlation analyses and machine learning models suggests that fatigue is not merely a symptomatic problem but a central determinant of quality of life. This finding supports the need for multidisciplinary approaches to fatigue management to be prioritized in rehabilitation programmes.

Functional mobility assessed using the TUG test showed a significant negative association with quality of life in the present study. Specifically, longer TUG completion times were moderately correlated with lower MSQoL-54 physical, mental, and total scores, indicating that reduced functional mobility is linked to poorer perceived quality of life among individuals with MS. These findings suggest that even relatively simple functional mobility measures may reflect broader limitations affecting daily activities and participation. Impaired mobility can reduce independence in daily living, limit community participation, and increase the risk of falls, which may collectively contribute to diminished physical and psychological well-being.^[30]^ Previous research has similarly reported that mobility impairments and balance deficits are important contributors to reduced health-related quality of life in people with MS.^[11]^ The moderate yet consistent correlations observed in our study further support the clinical relevance of the TUG test as a practical and objective indicator of functional status that may help identify patients at greater risk of poor quality of life.

In the present study, although depression level showed a strong negative correlation with quality of life, it had a relatively lower weight in the final Lasso model. This finding can be explained by the fact that the effect of depression on quality of life may partially overlap with physical variables such as disability and fatigue, or may manifest indirectly through these variables. In individuals with MS, psychological symptoms often coexist with physical limitations, and these factors tend to interact with each other rather than operate independently. For example, increased disability and fatigue may lead to reduced participation in daily and social activities, which in turn may exacerbate depressive symptoms and further contribute to a decline in perceived quality of life. Therefore, when multiple interrelated variables are simultaneously included in predictive models such as Lasso regression, the relative contribution of depression may appear lower because part of its explanatory effect is already captured by closely related clinical variables. Similarly, the literature reports that the impact of psychological factors on quality of life interacts with physical functional impairment and may vary depending on whether these factors are evaluated independently or within multivariable models.^[15,31]^ These findings suggest that quality of life in MS is shaped by a complex interplay between psychological and physical factors, and that comprehensive clinical assessment should consider both domains together rather than addressing them in isolation.

In the present study, although disease duration was not significantly associated with quality of life in the univariate analyses, it contributed positively in the multivariate machine learning model. This discrepancy may be explained by the fact that the influence of disease duration becomes more apparent when it is evaluated together with other clinical and functional variables rather than in isolation. In chronic conditions such as MS, individuals who have lived with the disease for a longer period may gradually develop coping strategies, adjust their expectations, and adapt their daily routines to their functional limitations. Over time, patients may also gain greater experience in symptom management and disease self-management, which can help mitigate the negative impact of the disease on perceived quality of life. As a result, quality of life may not necessarily decline in parallel with increasing disease duration. Instead, it may be more closely associated with the individual’s current functional status, symptom burden, and psychological adjustment rather than the length of time since diagnosis.^[32,33]^ Therefore, these findings suggest that clinical assessments focusing on present functional capacity and symptom severity may provide a more accurate understanding of quality of life in individuals with MS than disease duration alone.

The important contribution of the present study was the comparative evaluation of multiple machine learning algorithms, extending beyond traditional statistical approaches. The results indicate that more complex and non-linear models, such as Random Forest and XGBoost, did not outperform linear and regularized models in this sample. This finding may be related to the relatively small sample size and the high level of correlation among predictor variables, conditions under which regularization-based approaches such as Lasso and Elastic Net are known to perform particularly well. These methods are designed to handle multicollinearity and reduce model complexity by selecting the most relevant predictors, thereby improving model stability and predictive performance. In addition, the interpretability of linear and regularized models represents an important advantage in clinical research and practice, as it allows researchers and clinicians to more easily understand the relative contribution of individual variables to the predicted outcome.

Several limitations of this study should be considered when interpreting the findings. First, the cross-sectional design of the study limits the ability to draw causal inferences regarding the relationships between clinical variables and quality of life. Longitudinal studies are needed to better understand how these factors interact over time and influence changes in quality of life in individuals with MS. Second, although internal validation was performed using repeated five-fold cross-validation, the predictive models were not externally validated in an independent dataset. This limits the generalizability and clinical applicability of the findings, particularly considering the single-center design and relatively small sample size. Therefore, future studies should validate the proposed models in larger, independent, and multicenter cohorts to confirm their robustness and transportability across different clinical settings. Third, although several important clinical and psychological variables were included, other potentially relevant factors such as cognitive function, social support, physical activity levels, and medication use were not evaluated. Cognitive impairment is a common symptom in individuals with MS and has been reported to influence several aspects of daily functioning and overall quality of life; therefore, the absence of cognitive assessments may limit a more comprehensive understanding of the factors affecting quality of life in this population. Future studies should employ larger, independent, multicenter, and longitudinal designs and incorporate additional clinical variables such as cognitive function and physical activity to further improve the accuracy, robustness, and generalizability of machine learning models predicting quality of life in MS.

## 5. Conclusion

In conclusion, the present study showed that quality of life in individuals with MS was strongly associated with disability, fatigue, and functional mobility within the study sample. Among the evaluated machine learning models, Lasso regression demonstrated the best predictive performance and provided an interpretable ranking of multidimensional predictors. These findings suggest that machine learning approaches may be useful for exploring complex relationships between clinical, functional, and psychological factors related to quality of life in MS. However, because the models were internally validated only and were not tested on an independent external dataset, their generalizability and direct clinical applicability should be interpreted with caution. Future studies with larger, independent, and multicenter cohorts are needed to externally validate these models before they can be recommended for personalized rehabilitation planning or clinical decision-making.

## Acknowledgments

The authors would like to thank for their contributions to this study.

## Author contributions

**Conceptualization:** Aysu Yetiş, Mehmet Canli, İrem Canli, Hikmet Kocaman, Nazim Tolgahan Yildiz, Selcen Duran.

**Data curation:** Aysu Yetiş, Selcen Duran.

**Formal analysis:** Hasan Yildirim.

**Investigation:** Aysu Yetiş.

**Methodology:** Aysu Yetiş, Mehmet Canli, Hasan Yildirim.

**Resources:** İrem Canli.

**Software:** Aysu Yetiş, Hasan Yildirim.

**Validation:** Mehmet Canli, Nazim Tolgahan Yildiz.

**Visualization:** Aysu Yetiş, Mehmet Canli, İrem Canli, Hikmet Kocaman, Nazim Tolgahan Yildiz.

**Writing – original draft:** Aysu Yetiş, Mehmet Canli, İrem Canli, Hikmet Kocaman, Hasan Yildirim, Nazim Tolgahan Yildiz, Selcen Duran.

**Writing – review & editing:** Aysu Yetiş, Mehmet Canli, İrem Canli, Hikmet Kocaman, Hasan Yildirim, Nazim Tolgahan Yildiz, Selcen Duran.
